# The Immunological Enhancement Activity of Propolis Flavonoids Liposome *In Vitro* and *In Vivo*

**DOI:** 10.1155/2014/483513

**Published:** 2014-10-14

**Authors:** Yang Tao, Deqing Wang, Yuanliang Hu, Yee Huang, Yun Yu, Deyun Wang

**Affiliations:** ^1^Institute of Traditional Chinese Veterinary Medicine, College of Veterinary Medicine, Nanjing Agricultural University, Nanjing 210095, China; ^2^College of Life and Environment Science, Huangshan University, Huangshan 245041, China

## Abstract

The aim of this study was to investigate and assess the effects of propolis flavonoids liposome imposed on the immune system by comparing it to propolis flavonoids and blank liposome. *In vitro*, the effects of the above drugs on macrophages were assessed by measuring the phagocytic function and cytokine production. *In vivo*, the immunological adjuvant activity of propolis flavonoids liposome was compared with those of propolis flavonoids and blank liposome. The results showed that *in vitro* propolis flavonoids liposome can significantly enhance the phagocytic function of macrophages and the release of IL-1*β*, IL-6, and IFN-*γ*. In addition, subcutaneous administration of propolis flavonoids liposome with ovalbumin to mice could effectively activate the cellular and humoral immune response, including inducing higher level concentrations of IgG, IL-4, and IFN-*γ* in serum and the proliferation rates of splenic lymphocytes. These findings provided valuable information regarding the immune modulatory function of propolis flavonoids liposome and indicated the possibility of use of propolis flavonoids liposome as a potential adjuvant.

## 1. Introduction

Propolis is a complex mixture processed by honeybees from the resins collected from buds, leaves, and exudates of different plants, such as poplar, birch, horse chestnut, alder, beech, and conifer trees. It has attracted researchers' interest and also extensively appeared in the composition column as an additive in health foods, beverages, and nutritional supplements for improvement of health and prevention of several diseases because of its biological and pharmacological properties, including immunomodulatory, antitumor, anti-inflammatory, antioxidant, and antibacterial [1–6]. Over 150 constitutions were contained in propolis, such as polyphenols (flavonoids, phenolic acids, and their esters), terpenoids, steroid, and acids, but vary in the geographical and botanical origins [7]. Propolis from China contains many flavonoids [1] and flavonoids are thought to account for much of the biological and pharmacological activities. However, it makes propolis flavonoids difficult to be utilized because of the poor solubility in water.

Liposomes are microscopic vesicles composed of membrane-like lipid bilayers surrounding aqueous compartment. The lipid layers are comprised mainly of phospholipids [8]. Propolis flavonoids, mixes of various hydrophobic compounds, can be intercalated into the lipid bilayer and ultimately dissolved in the aqueous solution. Most importantly, liposomes are proved to be safe and well tolerated, as demonstrated through the extensive researches and applications of liposome-based anticancer [9] and anti-infective drugs [10]. In addition, effective active targeting and evidently prolonged resident time in blood can be purposefully achieved after specific modification of liposomes as drug delivery systems [11]. Accidentally, the oxidation process of phospholipids can be delayed due to the antioxidant effect exerted by propolis.

In addition, the research and development of liposomes as adjuvant has greatly intensified in the last 10 years, which exhibits advantages of elicitation of both humoral mediated immunity and cell mediated immunity and others described above [12–14]. Similarly, the vaccine containing propolis demonstrated a faster, more efficient, and more durable immune response than nonformulated one and furthermore less toxic than white oil [15]. Therefore, if propolis flavonoids are encapsulated with liposome, not only the water solubility of propolis flavonoids will be enhanced, but also they maybe have synergistic effect.

In this study,* in vitro*, the effect of the propolis flavonoids liposome on phagocytic activity and cytokine secretion of macrophages was measured. The effect of the propolis flavonoids liposome on the humoral mediated immunity and cell mediated immunity was compared with propolis flavonoids, liposome, and Freund's Complete Adjuvant (FCA). The aim of this strategy is to investigate whether immunological enhancement activity of propolis flavonoids and liposome formulations can further enhance or modulate the immune response against OVA vaccine compared with the adjuvant alone.

## 2. Materials and Methods

### 2.1. Preparation of Propolis Flavonoids Liposome

Propolis was purchased from Dahua Chinese traditional medicine company in Nanjing, Jiangsu province. Propolis flavonoids were prepared in our laboratory Briefly, propolis was extracted with 95% ethanol for three times and the ethanol solution was retrieved. Then, the precipitation was extracted with ethyl acetate for three times and then the ethyl acetate was retrieved. Finally, the precipitation was dried in vacuum and propolis flavonoids were obtained. Propolis flavanoids are a complex mixture which mostly contains rutin, myricetin, quercetin, kaempferol, apigenin, pinocembrin, chrysin, and galanigin. Propolis flavanoids was purchased from Dahua Traditional Chinese Medicine Company in Nanjing, Jiangsu province, China. The content of rutin in the propolis flavanoids was measured by UV-spectrophotometric method and the contents of chrysin and galanigin in the propolis flavanoids were measured by HPLC (Waters 2695, water 2489 UV/Visible Detector, waters Symmetry C18 Column, 100 Å, 5 *µ*m, 4.6 mm × 250 mm, 1/pkg. Milford, Massachusetts, USA). The rutin, chrysin, and galanigin contents of propolis flavonoids used in our experiments were, respectively, 50.67%, 6.50%, and 22.68%, which was in accord with the standard of Chinese Pharmacopoeia. The propolis flavonoids liposome (the average size of propolis flavonoids liposome was about 100 nm and the encapsulation efficiency of propolis flavonoids liposome was 91%) was prepared with the ethanol injection method according to our previous research [16].

### 2.2. Assay for Macrophages Activation by Propolis Flavonoids Liposome

#### 2.2.1. Peritoneal Macrophage Preparation

Peritoneal macrophages were isolated with minor modifications, as described in previous report [17]. In brief, peritoneal macrophages were harvested from ICR mice (4 weeks old) 2 days after an intraperitoneal injection of 1 mL 6% starch-broth medium. ICR mice were sacrificed and about 5 mL of PBS was injected into the abdominal, then the abdomen was massaged gently for 3 min, and the PBS was drawn back with peritoneal fluid. After centrifugation at 1500 rpm for 10 min, the macrophages were collected and washed twice with PBS. Peritoneal macrophages were resuspended and diluted to 2.5 × 10^6^ mL^−1^ with RPMI-1640 with fetal bovine serum. The cells were transferred to 24-well plates (Costar, Corning, NY, USA) and incubated to adhere for 4 h in a humid atmosphere (Thermo Scientific, Waltham, MA, USA) with 5% CO_2_ at 37°C. Then remove nonadherent cells and wash gently twice with RPMI 1640 medium.

#### 2.2.2. Phagocytosis Assay

The effects of propolis flavonoids liposome, propolis flavonoids, and blank liposome on phagocytic function of macrophages were assessed by Vybrant Phagocytosis Assay Kit (Molecular Probes, Inc, Eugene, OR, USA) and ultimately the total mean fluorescence intensity (MFI) was determined by flow cytometry (BD FACSVerse, San Jose, CA). Briefly, peritoneal macrophages (2.5 × 10^6^/mL in RPMI complete medium, 2 mL per well) were exposed to different concentrations of propolis flavonoids liposome (60 *μ*g/mL, 30 *μ*g/mL, and 15 *μ*g/mL; according to previous results, the 50% cytotoxic concentration of the propolis flavonoids was 181.30 *μ*g/mL and the maximal safety concentration of the propolis flavonoids 60 *μ*g/mL), propolis flavonoids (60 *μ*g/mL, 30 *μ*g/mL, and 15 *μ*g/mL), blank liposomes, LPS (being from* Escherichia coli* 055:B5, 1 *μ*g/mL, purity > 97%, Sigma, St. Louis, MO, USA), and RPMI-1640 medium for 48 h. Then the supernatants were removed from the 24-well plates carefully and 600 *µ*L of the prepared fluorescent BioParticle suspensions was added to all the wells. After incubation for 2 hours at 37°C in 5% CO_2_, the BioParticle loading suspensions were removed and 600 *µ*L of the prepared trypan blue suspensions was added for 1 minute at room temperature. The excess trypan blue suspensions were removed and 0.5 mL 0.25% trypsin was added to each sample for 2 minutes. Finally, the macrophages were washed twice with PBS, fixed with 600 *µ*L 4% paraformaldehyde, and examined by flow cytometry.

#### 2.2.3. Measurement of Cytokine Production

After peritoneal macrophage was prepared as Section 2.2.1, propolis flavonoids liposome at series of concentrations (60 *μ*g/mL, 30 *μ*g/mL, 15 *μ*g/mL), propolis flavonoids (60 *μ*g/mL, 30 *μ*g/mL, and 15 *μ*g/mL), blank liposomes, and RPMI-1640 medium were, respectively, added in a final volume of 150 *μ*L per well, four wells each concentration. The plates were incubated in a humid atmosphere at 37°C with 5% CO_2_ for 48 h. The supernatant was harvested and the tumor necrosis factor alpha (TNF-*α*), interleukin 1 beta (IL-1*β*), interleukin 6 (IL-6), interleukin 12 (IL-12), and interferon-gamma (IFN-*γ*) production were analyzed by using the Quantikine Mouse cytokines ELISA kits (R&D Systems, Inc, Minneapolis, MN, USA), respectively, according to the manufacturer's instructions.

### 2.3. Adjuvant Activity* In Vivo*

#### 2.3.1. Animal Treatment Protocol

The same quantity of female and male ICR mice (4 weeks old) were purchased from Comparative Medicine Centre of Yangzhou University and acclimatized for 7 days prior to use. The mice were maintained under controlled conditions at temperature of 24 ± 1°C, humidity of 50 ± 10%, and a 12/12-h light–dark cycle with free access to food and water. Each mouse was used once and treated in accordance with the National Institutes of Health guide lines for the care and use of laboratory animals.

#### 2.3.2. Immunization

Mice were injected subcutaneously in the dorsal skinfold on days 0 and day 14 with 0.5 mL propolis flavonoids liposome (0.5 mg/mL), propolis flavonoids (0.5 mg/mL), FCA adjuvant, and blank liposome, each formulation containing OVA (200 *μ*g). On weeks 1, 2, 3, 4, 5, and 6 after the first vaccination, mice were euthanized and the peripheral blood samples and the splenic lymphocyte were collected to determine the concentrations of OVA-specific IgG, IL-4, IFN-*γ*, and spleen lymphocyte proliferation.

#### 2.3.3. Measurements of Serum IL-4 and IFN-*γ*

Collected peripheral blood samples were placed in incubator (37°C) for 2-3 h and then were centrifuged at 10,00 rpm at 4°C for 10 min to collect the serum. The concentrations of IL-4 and IFN-*γ* in the serum were determined with ELISA kit (BD Biosciences, Franklin Lakes, New Jersey, USA) in accordance with manufacturer's instructions.

#### 2.3.4. Measurements of OVA-Specific IgG

OVA-specific IgG in serum was detected by an indirect ELISA [18–20]. In brief, microtiter plate wells were coated with 100 *μ*L OVA solution (coating buffer-PBS containing 0.4 *μ*g OVA) and incubated for 12 h at 4°C. The wells were washed three times with washing buffer containing 0.12% (w/w) Tris and 0.85% (w/w) NaCl in deionized water and blocked with 1% (v/v) gelatin/PBS at 37°C for 1 h. After three times of washing, 100 *μ*L of a series of diluted serum samples was added into triplicate wells. The plates were incubated for 2 h at 37°C, followed by three times' washing. At this point, 100 *μ*L of peroxidase (HRP)-conjugated AffiniPure goat anti-mouse IgG (H + L) diluted to 1 : 5000 with PBS containing 0.05% (v/v) Tween 20 (PBST) was dispensed into each well and incubated at 37°C for 1 h. After three times of washing, 100 *μ*L of 3′,3′,5′,5′-tetramethyl benzidine (TMB, Tiangen Biotech Co., Ltd) was added to each well. The plates were in the dark at 37°C for 15 min. The reaction was terminated by the addition of 100 *μ*L of stopping buffer (2 mol/L H_2_SO_4_ solution). The absorbance was measured at 450 nm. The standard curve of IgG, on which the concentrations of the standard IgG were as abscissa and the relative *A*_450_ as ordinate, was established. The concentration of IgG of each sample serum was calculated according to the standard curve.

#### 2.3.5. Splenic Lymphocyte Proliferation Assay

Splenic lymphocyte proliferation was measured as described [21, 22]. The spleen cells were seeded into 96-well culture plates and phytohemagglutinin (PHA, purity > 97%, Sigma, 10 *μ*g/mL) or lipopolysaccharide (LPS, being from* Escherichia coli* 055:B5, 1 *μ*g/mL, purity > 97%, Sigma, St. Louis, MO, USA) was added to stimulate T or B lymphocyte proliferation. Then 20 *μ*L of RPMI-1640 medium was added into the left wells as the control. After 44 h incubation at 37°C, 30 *μ*L of MTT solution (5 mg/mL) was added to each well and incubated for an additional 4 h. The supernatant was removed, and then the formed formazan salts were dissolved by 100 *μ*L of DMSO in shaking plates for 10 min. Absorbance was measured at 570 nm using an ELISA reader (Multiskan FC Microplate photometer, Thermo scientific, USA). The proliferation rate (%) was calculated according to the following equation:

(1) $$\begin{array}{c} \text{Proliferation}\;\text{rate}\left(\%\right)=\frac{\left[{\overset{-}{A}}_{\left(\text{drug}\;\text{group}\right)}-{\overset{-}{A}}_{\left(\text{control}\;\text{group}\right)}\right]}{{\overset{-}{A}}_{\left(\text{control}\;\text{group}\right)}}. \end{array}$$

### 2.4. Statistical Analysis

Data are expressed as mean ± standard errors (S.E.). Duncan and LSD's multiple range test were used to determine the difference among groups. *P* values of less than 0.05 were considered to be statistically significant.

## 3. Results

### 3.1. Propolis Flavonoids Liposome Enhanced the Phagocytosis Function of Macrophages

**Figure 1:**
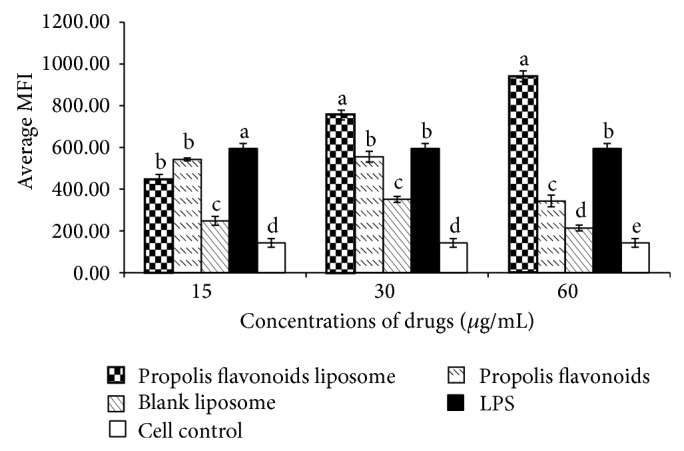
The MFI of the phagocytosis response on each group. LPS, three different concentrations of propolis flavonoids liposome, propolis flavonoids, and blank liposome were added to peritoneal macrophages. The macrophages were handled with Vybrant Phagocytosis Assay Kit after 48 h incubation. The phagocytosis function was finally assessed by determining the average MFI. Values are mean ± SEM, *n* = 6. ^a–e^ The superscripts without the same letters above the columns differ significantly (*P* < 0.05) from each other in the same concentration group.

The process of phagocytic is essential for macrophage as important antigen present cells and the first line of defense. The effects of the propolis flavonoids liposome, propolis flavonoids, blank liposome, and LPS on macrophages were assessed. As shown in Figure 1, phagocytic function of macrophages after adding propolis flavonoids liposome was significantly enhanced and tended to be dose-dependent. The results showed that not only propolis flavonoids liposome but also propolis flavonoids, blank liposome, and LPS enhanced the phagocytic function of macrophages.

### 3.2. Propolis Flavonoids Liposome Stimulated Cytokines Production of Murine Peritoneal Macrophages

**Figure 2:**
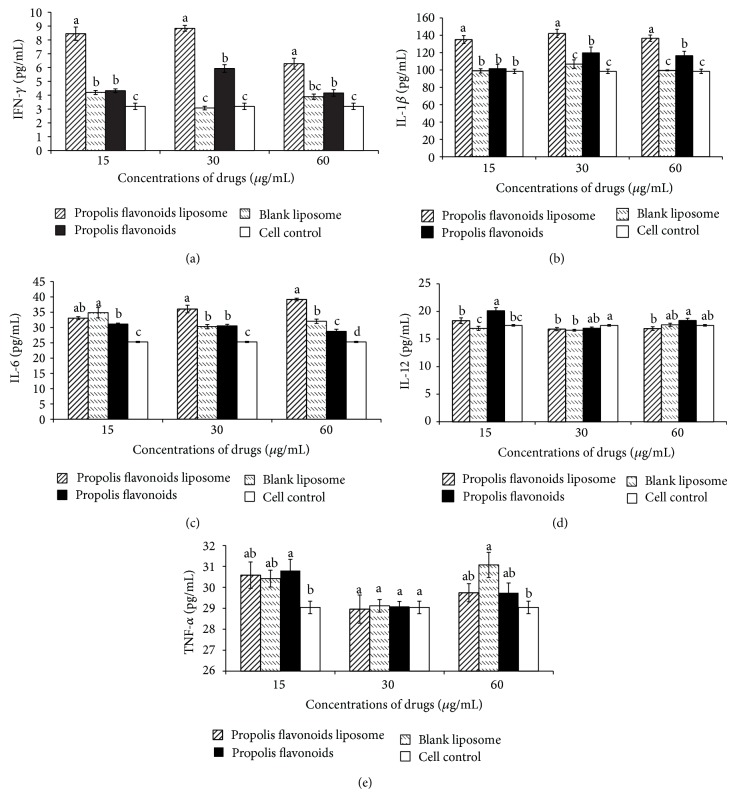
The cytokines production of the peritoneal macrophages on each group (pg/mL). The effects of propolis flavonoids liposome, propolis flavonoids, and blank liposome on peritoneal macrophages were assayed. (a) TNF-*α*, (b) IL-1*β*, (c) IL-6, (d) IL-12, and (e) IFN-*γ*. Values are mean ± SEM, *n* = 6. ^a–d^ The superscripts without the same letters above the columns differ significantly (*P* < 0.05) from each other in the same concentration group.

The murine peritoneal macrophages were stimulated with propolis flavonoids liposome for 24 h and the cytokines contents in the culture supernatant were measured by ELISA. As a result, propolis flavonoids liposome (60 *μ*g/mL, 30 *μ*g/mL, and 15 *μ*g/mL) could significantly increase the production of the IFN-*γ*, which was a significant difference between with propolis flavonoids and blank liposome (Figure 2(a)). The production of the IL-1*β* of propolis flavonoids liposome (60 *μ*g/mL, 30 *μ*g/mL, and 15 *μ*g/mL) groups was significantly higher than those of propolis flavonoids and blank liposome (Figure 2(b)). The IL-6 concentrations of propolis flavonoids liposome groups in 30 *μ*g/mL and 60 *μ*g/mL were significantly higher than those in other groups (*P* < 0.05) (Figure 2(c)). However, they failed to reproduce the similar trends above in terms of IL-12 and TNF-*α* (Figures 2(d) and 2(e)).

### 3.3. Propolis Flavonoids Liposome Increased the Splenic Lymphocyte Proliferation

**Figure 3:**
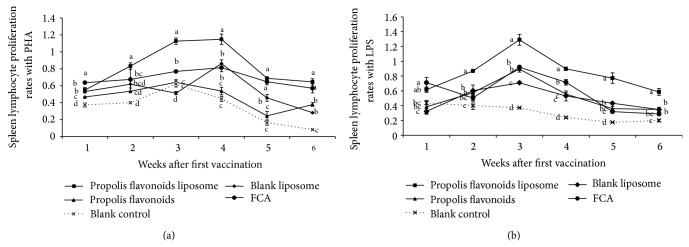
The dynamic change of spleen lymphocyte proliferation in synergistic stimulation of drugs with PHA (*A*_570_) (a) and LPS (*A*_570_) (b) after vaccination. ICR mouse was inoculated twice on days 0 and 14 with OVA encapsulated in propolis flavonoids liposome, blank liposome, and Freund's Complete Adjuvant or mixed in propolis flavonoids and the proliferation rates of spleen lymphocyte mix with PHA or LPS were measured on weeks 0, 1, 2, 3, 4, 5, and 6 after first vaccination. Values are mean ± SEM, *n* = 4 mice/group. ^a–d^ The superscripts without the same letters near the curves differ significantly (*P* < 0.05) from each other in the same week group.

To compare the ability of propolis flavonoids liposome, propolis flavonoids, liposomes, and FCA to induce cellular immune response, the splenic lymphocyte proliferation of vaccinated mice was quantified after weeks 1, 2, 3, 4, 5, and 6 of immunization. The result as seen in Figures 3(a) and 3(b) is as follows: significantly higher magnitude of splenic proliferation with PHA was obtained in propolis flavonoids liposome group from week 2 to week 4 after first vaccination compared with propolis flavonoids, blank liposome, FCA, and blank control groups (*P* < 0.05). In addition, from week 2 to week 6 after first vaccination, significantly higher magnitude of splenic proliferation with LPS was obtained in propolis flavonoids liposome group compared with propolis flavonoids, blank liposome, FCA, and blank control groups (*P* < 0.05).

### 3.4. Propolis Flavonoids Liposome Enhanced the Production of IgG

**Figure 4:**
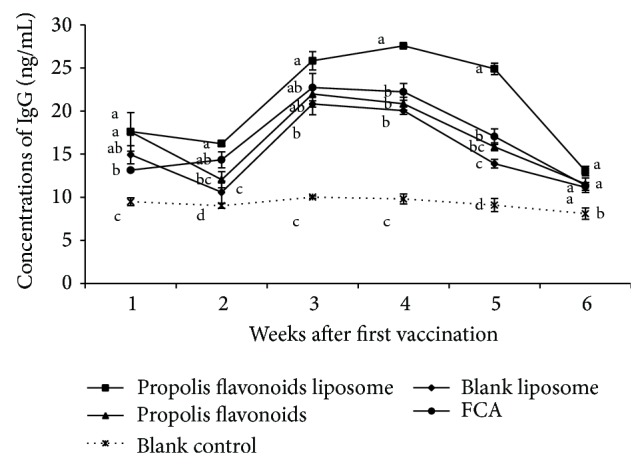
The dynamic change of IgG after vaccination. ICR mouse was inoculated twice on days 0 and 14 with OVA encapsulated in propolis flavonoids liposome, blank liposome, and Freund's Complete Adjuvant or mixed in propolis flavonoids. The concentrations of IgG in serum were measured on weeks 0, 1, 2, 3, 4, 5, and 6 after first vaccination. Values are mean ± SEM, *n* = 4 mice/group. ^a–d^ The superscripts without the same letters near the curves differ significantly (*P* < 0.05) from each other in the same week group.

The humoral immune response against most exogenous antigens can be evaluated by determining the immunoglobulin in blood, especially IgG, the major constitution. As shown in Figure 4, a significantly greater concentration of IgG was observed in mice vaccinated with propolis flavonoids liposome (*P* < 0.05) as compared to propolis flavonoids and liposome. Propolis flavonoids liposome was also more efficient (*P* < 0.05) at inducing the concentration of IgG compared to mice vaccinated with FCA on weeks 4 and 5 after first vaccination.

### 3.5. Propolis Flavonoids Liposome Promoted the IFN-*γ* and IL-4 Productions

**Figure 5:**
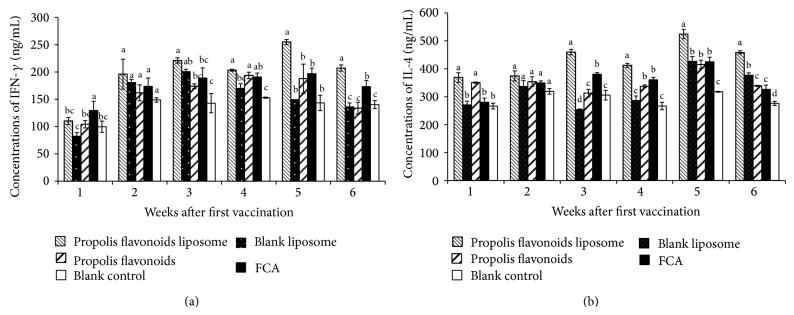
The dynamic change of concentrations of IFN-*γ* and IL-4 after vaccination. On weeks 0, 1, 2, 3, 4, 5, and 6 after first vaccination, the concentrations of IFN-*γ* and IL-4 in serum were determined by ELISA kits. Values are mean ± SEM, *n* = 4 mice/group. ^a–d^ The superscripts without the same letters above the columns differ significantly (*P* < 0.05) from each other in the same concentration group.

The massive secretion of cytokines by T-helper cells was essential in the process of specific immunity. IFN-*γ* and IL-4, as Th1 cytokine and Th2 cytokine, were assessed. The concentrations of IFN-*γ* in mouse vaccinated with propolis flavonoids liposome were significantly higher than mouse vaccinated with propolis flavonoids, liposome, and FCA on weeks 5 and 6 after primary vaccination (*P* < 0.05) (Figure 5(a)). Similarly, propolis flavonoids liposome resulted in a significantly higher amount of IL-4 than others on weeks 3 to 6 after first vaccination (*P* < 0.05) (Figure 5(b)).

## 4. Discussion

An ideal vaccine adjuvant is a component that not only can improve the effectiveness of vaccines by inducing robust immune responses [23] but also should be safe enough during vaccination. Therefore, the success of vaccines may lie in their association with selected adjuvants, especially to the innovative vaccines with poor immunogenicity. As various vaccines are developed, many types of adjuvants are also sought out and undergo evaluation in terms of security and availability. For example, although alum is widely used as a vaccine adjuvant, its role in nephrotoxicity, Alzheimer's disease, subcutaneous reaction, epitope modification, its inability to induce cellular response, and its limited effect on polysaccharide antigens highlighted the need for new adjuvants. In addition, despite the fact that Freund's Complete Adjuvant can lead to immune responses characterized by a mixed Th1/Th2 response and is useful for inducing cell-mediated response, its high toxicity, potential adverse reaction, and high cost make it unfit to be used as a vaccine adjuvant for humans [24–27].

In view of shortages of synthetic compounds, some natural products become fertile sources for new medicines [28]. The* in vivo* preclinical investigations highly recommend further applications [15]. The immunomodulatory effects of natural substances, such as propolis, ginsenoside, and QS21, have been considered as alternative adjuvant therapies in the treatment of various diseases [29, 30]. When used as a vaccine adjuvant, propolis has been shown to increase the safety of the associated vaccine, in addition to increasing its protective index, eliciting a higher antibody titer, eliciting high and persistent mucosal immunity, enhancing the cellular response, offering a higher phagocytic activity, increasing leukocytic reaction, promoting peripheral lymphocytes proliferation, extending vaccine protection, inducing early protection, reducing the optimum dose concentration, and enhancing nonspecific immunity regardless of the types of vaccine preparation [31, 32]. Moreover, these effects were usually correlated to the flavonoids content [33, 34]. However, propolis flavonoids difficultly dissolve into water. Liposomes, biodegradable and essentially nontoxic vehicles, can encapsulate both hydrophilic and hydrophobic materials [35]. In addition, liposomes are themselves the immunological adjuvant and have been confirmed and extended to include a wide range of antigens from bacteria, protozoa, viruses, tumors, and spermatozoa [36, 37]. Therefore, if propolis flavonoids are encapsulated with liposome, not only solubility of propolis flavonoids will be increasingly promoted, but also the immunological adjuvant will be synergistic. In this study, the propolis flavonoids were encapsulated with liposome and we compared the immunological enhancement activity with propolis flavonoids* in vitro* and* in vivo*.

As mononuclear phagocytic cells derive from peripheral blood monocytes and are resident in most tissues, macrophages function as professional antigen presenting cells (APCs) and as effector cells in humoral and cellular immunity. They act as the bridge between the innate immune system and the adapt immune system by differentiating into cells to exert diverse functions after being activated by different stimuli or combination of stimuli [38, 39]. Consequently, propolis flavonoids liposome, as stimuli, was incubated with peritoneal macrophage. The phagocytic capability of macrophages was significantly enhanced when exposed to propolis flavonoids liposome as shown in Figure 1. The process of phagocytosis is essential for macrophage to clear invades and present antigens to effective T cells. In addition, significantly greater concentrations of IL-1*β*, IL-6, and IFN-*γ* with adding PFL were found as compared to adding PF or liposome. However, the effects of propolis flavonoids liposome are not always dose-dependent. The effect of propolis flavonoids liposome on IL-6 (Figure 2(c)) appeared to be dose-dependent, whereas the releases of IL-1*β* and IFN- *γ* (Figures 2(b) and 2(a)) were more obvious on the middle concentration of propolis flavonoids liposome. In addition, propolis flavonoids liposome could not obviously promote the secretion of IL-12 and TNF-*α*. That also indicated that the propolis flavonoids liposome only affected secretion of partial cytokine. IL-1*β*, IL-6, and IFN-*γ* can promote the immune responses, including strikingly enhancing antigen-driven responses of CD4 and CD8 T cells [40], determining the path of T cell differentiation, inducing B cells to differentiate into plasma cells, and driving the production of Th1 cells. It demonstrated that the propolis flavonoids liposome, similar to PAMP, could be recognized by innate immune system through interacting with the macrophages and the results might be explained to be related to the activation of TLRs signal paths [41, 42].

*In vivo*, results showed that a substantial effect of PFL on the IgG concentration was seen. At later period of immunization (from 4 to 5 weeks), the effect of propolis flavonoids liposome was significantly superior to PF and liposomes, which indicated that the PF with encapsulated liposomes was controlled release. Moreover, significant difference was seen between propolis flavonoids liposome and FCA. From week 2 to week 4, the spleen lymphocyte proliferation rates of propolis flavonoids liposome group were significantly higher than those of other groups. It indicated that cellular immunity response could be boosted when propolis flavonoids liposome served as adjuvant and high levels of IFN-*γ* in propolis flavonoids liposome group were also improved. High levels of IL-4 and IgG in propolis flavonoids liposome group showed that propolis flavonoids liposome could efficiently induce the humoral immunity responses.

In a word, propolis flavonoids liposome not only could promote the phagocytic capability and the cytokine production of murine peritoneal macrophages* in vitro*, but also could enhance the humoral immunity response and cellular immunity response. Moreover, in contrast to FCA and alum, propolis flavonoids liposome was nontoxic and had few adverse reactions and superior effect, which is fitter to be used as vaccine adjuvant.

## Abbreviations

OVA: Ovalbumin
DMSO: Dimethyl sulfoxide
PHA: Phytohemagglutinin
FCA: Freund’s Complete Adjuvant
LPS: Lipopolysaccharide
MTT: 3-(4,5-Dimethylthiazol-2-yl)-2,5-diphenyltetrazolium bromide
PBS: Phosphate buffered saline
ELISA: Enzyme linked immunosorbent assay
IgG: Immunoglobulin G
IFN-*γ*: Interferon-*γ*
IL-12: Interleukin-12
IL-6: Interleukin-6
IL-1*β*: Interleukin-1*β*
IL-4: Interleukin-4
TNF-α: Tumor necrosis factor-α.
